# Key technologies of robotic arm motion control based on compound control and improved SCSO

**DOI:** 10.1371/journal.pone.0328691

**Published:** 2025-07-22

**Authors:** Kuo Hu

**Affiliations:** School of Mechanical and Power Engineering, Zhengzhou University, Zhengzhou, China; Yalova University, TÜRKIYE

## Abstract

As modern industrial automation advances towards intelligence and flexibility, robotic arms are widely used in precision manufacturing, intelligent assembly, and medical surgery. To meet the high-precision motion control demand, this study proposes a robotic arm motion control technology integrating composite control and an improved sandcat swarm optimization algorithm. The algorithm is enhanced by introducing the Iterative chaotic iterative mapping and sparrow warning mechanism. The composite control strategy combines visual guidance with BP-PID control. Experimental results show Joint 1 has an average angle of about 50° and an average angular velocity of 0°/s. Joint 2 has an angle of about −80°, with an M-shaped angular velocity curve and large angular acceleration fluctuations, reaching a minimum angular jerk of −90/s³. Joint 3 has an angle of approximately 0°, with a W-shaped angular velocity curve and a maximum angular acceleration of 17°/s². The findings indicate the proposed technology accurately matches the kinematic and dynamic characteristics of robotic arm movements, ensuring precise joint motion trajectories and effectively suppressing shocks and vibrations. This research offers new ideas and methods for robotic arm motion control technology, with significant theoretical and practical value.

## 1. Introduction

The performance benefits and drawbacks of robotic arms (RAs), the primary actuator of industrial automation in the rapidly evolving field of intelligent manufacturing, have a direct impact on the productivity and caliber of output [1]. In many complex industrial scenarios, such as precision assembly, high-risk environmental operations, etc., the motion control accuracy, response speed, and stability of the RA have put forward extremely stringent requirements [2]. Traditional RA motion control technology, in the face of increasingly complex tasks and dynamic changes in the working environment, exposed many limitations [3]. Although some intelligent control algorithms have improved the performance to a certain extent, they are prone to fall into local optimal solutions and cannot realize efficient optimization in the global scope [4].

The advantages of sand cat swarm optimization (SCSO), a new intelligent optimization technique, include its powerful global search capabilities and quick convergence. The method draws inspiration from the sand cat (SC)‘s ability to sense low-frequency noise, which allows it to locate its prey whether it is underground or on the ground [5]. However, SCSO also suffers from problems such as premature convergence in practical applications. Numerous professionals and academics have put up a variety of development solutions to address the premature convergence issue of SCSO. For example, the IMSCSO algorithm proposed by X. Li et al. significantly improved the algorithm’s global search capability (GSC) and convergence speed (CS) by introducing a low-frequency noise search strategy (LFNSS) and a spiral contraction walk strategy [6]. J. Li et al. significantly improved the overall performance of the algorithm by introducing an enhanced circular chaotic mapping and dynamic opposition learning strategy [7]. By adding an LFNSS and a spiral contraction walking strategy, together with a stochastic opposition learning and restart method, Y. Hu improved the algorithm’s GSC and CS even further [8]. The compound control algorithm is the key technology of the RA motion control system. The study and application of this technology can improve the performance of the control system, make the robot easy to operate, and achieve more accurate positional localization and more precise torque control [9]. In recent years, many researchers and scholars have carried out in-depth studies in this field and achieved a series of important results. Y. Zhang and C. Hua proposed a finite-time control method based on composite learning for robotic systems with output constraints. The experimental results indicated that the method could maintain good control performance under a variety of complex working conditions [10]. U. Javaid et al. designed a composite robust controller for the RA trajectory tracking (TT) problem. The results indicated that the composite robust controller was able to realize high precision TT in complex dynamic environments, providing a reliable technology for the application of robots in complex tasks [11]. A compound control scheme-based autonomous operation technique for underwater robots was proposed by M. Cai et al. The outcomes of the experiment demonstrated that even in the face of outside interference, the compound control scheme could achieve high-precision autonomous operation [12]. However, when dealing with complex working conditions such as multi-source interference coupling and rapid switching of task requirements in industrial sites, the existing methods still suffer from insufficient dynamic adjustment of control accuracy, limited response speed, and insufficient robustness. This indicates that there is still a large research gap to be filled in the field of RA motion control. Based on previous studies and in response to the above research gaps, this study proposes motion control technology for RAs that integrates improved SCSO and compound control. The aim is to enhance the accuracy and adaptability of RA motion control in complex industrial environments.

The innovations of this study are:(1) In the aspect of analyzing the motion trajectory of the RA, based on the traditional SCSO algorithm, the study introduces the Iterative chaotic iterative mapping and sparrow warning mechanism to enhance the global search ability and convergence speed (CS) of the algorithm. Meanwhile, the B-5-B hybrid polynomial interpolation function is used to further optimize the motion trajectory planning of RA, enabling it to exhibit higher continuity in complex tasks. (2) Regarding the RA’s motion trajectory control, the study proposes a composite control strategy that integrates visual guidance and back propagation (BP)-proportional-integral-derivative (PID) control. The position information of the RA is fed back in real time through visual guidance. The precise regulation ability of the BP neural network-PID controller enables high-precision control of the RA’s movement, ensuring the accuracy of its end position and the smoothness of the trajectory.

The contributions of this study are: (1) It proposes an improved sandcat swarm optimization algorithm that enhances the global search ability and convergence speed of the traditional algorithm. This improvement effectively addresses the shortcomings of existing methods in complex working conditions, making the motion control of RAs more accurate and adaptable. (2) By proposing the improved algorithm and composite control technology, this research not only offers new ideas and methods for the broad application of RAs in intelligent manufacturing but also holds significant theoretical and practical value. It paves the way for further advancements in the field of robotic automation and contributes to the development of intelligent manufacturing systems.

## 2. Methods and materials

To achieve precise control of the RA, the research conducts technological innovation from two aspects: algorithm improvement and control strategy. At the algorithmic level, the introduction of the iterative chaotic mapping and the sparrow alert mechanism endows SCSO with a stronger global search ability and faster CS, helping it to avoid falling into a local optimum. The sparrow alert mechanism accelerates the convergence process by simulating group collaboration. Based on this and the B-5-B mixed polynomial interpolation function, the trajectory planning of the RA is ensured to be continuous and stable. In terms of control methods, a composite control strategy integrating visual guidance and BP neural network-PID is constructed. The visual servo system provides real-time feedback on the end position, while the BP neural network dynamically optimizes the PID parameters. This solves the problem of traditional PID parameter adjustment relying on experience.

### 2.1 RA motion trajectory based on improved SCSO

If a precise control of RA motion is desired, its trajectory needs to be analyzed first. SCSO is an optimization algorithm based on bio-heuristic algorithm. The algorithm simulates the hunting behavior of SCs and the mechanism of group collaboration to achieve the solution of complex optimization problems. The study uses the efficient search mechanism of SCSO algorithm to determine the optimal trajectory of the RA under different working conditions. Assuming that the population size of SC is N , the initial position of the individual position of SC in the D -dimensional search space is shown in Equation (1).


$$X_{i}=(x_{i1},x_{i2},...,x_{iD}),i=1,2,...,N$$ (1)

In Equation (1), i denotes the individual. The auditory sensitivity range of the SC determines its exploratory ability, and the auditory sensitivity range R is shown in Equation (2).


$$R=R_{\max}-(\frac{R_{\max}-R_{\min}}{t_{\max}})\cdot t$$ (2)

**Fig 1:**
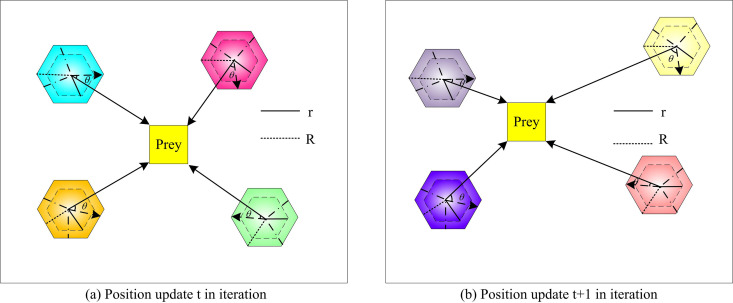
Location update in iteration.

In Equation (2), $R_{\max}$ denotes the maximum sensitivity range. $R_{\min}$ denotes the minimum sensitivity range. t is the current iteration number. $t_{\max}$ is the maximum number of iterations. The sensitivity range of the SC is set to be a hexagon. The position is updated as shown in Fig 1.

Fig 1(a) shows the schematic diagram of position update $X_{new}$. r denotes the sensitivity of each SC. At this time, ‖R‖ >1, the SC is in the exploration stage, and the individual position is updated according to the random angle θ and the sensitivity range R. The position update $X_{new}$ is displayed in Equation (3).


$$X_{new}=X_{i}+R\cdot \cos(\theta )\cdot \left|X_{rand}-X_{i}\right|$$ (3)

In Equation (3), $X_{rand}$ is the position of a randomly selected individual in the former population. cos(θ) denotes the simulated SC sensing prey direction through hearing. Fig 1(b) shows the schematic diagram of position update ${X_{new}}^{\prime }$. Currently, when ‖R‖ ≤1, the SC is in the developmental stage and adopts an aggressive behavior. The position update ${X_{new}}^{\prime }$ is shown in Equation (4).


$${X_{new}}^{\prime }=X_{best}-R\cdot \cos(\theta )\cdot \left|X_{best}-X_{i}\right|$$ (4)

In Equation (4), $X_{best}$ is the current global optimal solution (GOS). Stage switching is realized by adaptive adjustment of the sensitive range R. To avoid the solution exceeding the feasible domain, reflective boundary processing is used, as shown in Equation (5).


$$X_{ij}=\{\begin{array}{cc} {}*20cLB_{j}+(UB_{j}-LB_{j})\cdot rand & x_{ij}<LB_{j}\\ UB_{j}+(UB_{j}-LB_{j})\cdot rand & x_{ij}>UB_{j} \end{array}$$ (5)

In Equation (5), $LB_{j}$ is the lower bound of the j th dimension variable. $UB_{j}$ is the upper bound of the j th dimension variable. rand is a random number between 0 and 1. The study uses an iterative chaotic iterative mapping to further improve the GSC of SCSO. The chaotic iterative mapping is a chaotic system implemented by iterative means, involving one or more nonlinear functions, which are applied iteratively to generate chaotic sequences [13]. The Iterative-based chaotic iterative mapping $z_{k+1}$ is shown in Equation (6).


$$z_{k+1}=\sin(\frac{a\pi }{z_{k}})$$ (6)

**Fig 2:**
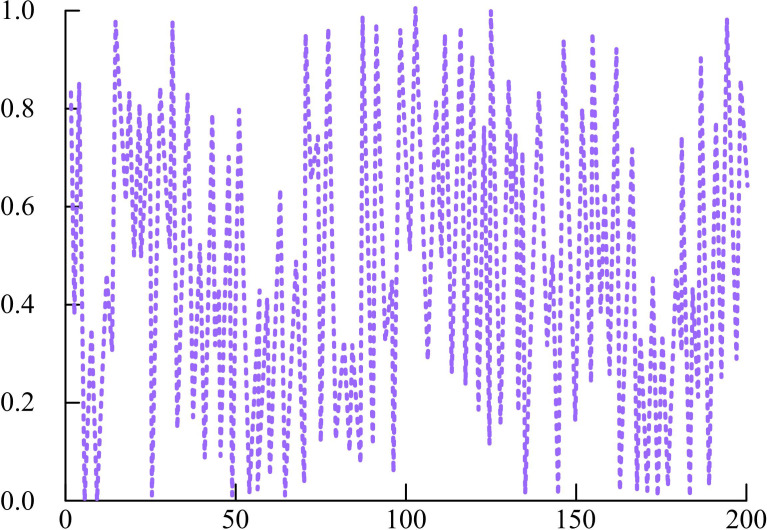
Chaotic iterative mapping diagram based on iterative.

In Equation (6), a denotes the control parameter. Assuming 200 iterative mappings and a of 0.7, the Iterative chaotic iterative mapping schematic is shown in Fig 2.

In Fig 2, the Iterative chaotic iterative mapping generates chaotic sequences by iteration in 200 iterations of mapping. Chaotic sequences show high sensitivity during the iteration process. That is, a small change in the initial conditions will lead to a huge difference in the results of the subsequent iterations, reflecting the initial condition sensitivity of the chaotic system. The chaotic sequence is able to traverse the entire definition domain during the iteration process, and the distribution is relatively uniform. It indicates that Iterative chaotic iterative mapping has good traversal and pseudo-randomness [14]. Chaotic sequences are useful in optimization algorithms to improve the GSC because of these characteristics, which aid the algorithm in better exploring the search space and avoiding local optimal solutions. To enhance the CS of SCSO, the study introduces the sparrow vigilance mechanism. The sparrow vigilance mechanism is a strategy that simulates the vigilance behavior of sparrow groups in nature. In the sparrow vigilance mechanism, the vigilant is responsible for monitoring potential threats in the environment and guiding the group to search effectively through specific behaviors [15]. In this case, the vigilant position update formula is shown in Equation (7).


$$X_{alert}^{t+1}=\{\begin{array}{cc} {}*20cX_{best}^{t}+\beta \cdot \left|X_{rand}^{t}-X_{best}^{t}\right| & f_{1}>f_{2}\\ X_{best}^{t}+K\cdot (\frac{X_{worst}^{t}-X_{best}^{t}}{\left\|X_{worst}^{t}-X_{best}^{t}\right\|+\rho }) & f_{1}=f_{2} \end{array}$$ (7)

In Equation (7), $X_{best}^{t}$ denotes the GOS vector in the t th generation population. $X_{rand}^{t}$ denotes the vector of randomly selected individuals in the t th generation. $X_{worst}^{t}$ denotes the worst solution vector in the t th generation population. β denotes the random perturbation coefficient obeying a standard normal distribution. K denotes directional escape coefficient. ρ denotes the very small constant, and the current global optimal fitness value (FV) is denoted by the symbol $f_{1}$. The current FV of an individual SC is denoted by the symbol $f_{2}$. In the RA trajectory control, SCSO is used to optimize the minimum energy consumption objective function of the joint space trajectory q(t), as shown in Equation (8).


$$\min\int_{t_{0}}^{t_{f}}{\left\|\tau (q,\dot{q},\ddot{q})\right\|}^{2}dt$$ (8)

In Equation (8), τ denotes the joint moment. q denotes the joint angular vector. $\dot{q}$ denotes the joint angular acceleration vector. $\ddot{q}$ denotes the joint angular acceleration vector. $t_{0}$ denotes the initial time of the trajectory. $t_{f}$ denotes the termination time of the trajectory. SCSO achieves optimal trajectory generation by adjusting the path point positions $q_{i}$. In robot joint trajectory planning, B-5-B mixed polynomial interpolation function is used in the study. The B-5-B mixed polynomial conducts research on the robot’s motion trajectory during the truss assembly process through piecewise polynomials, ensuring smooth and continuous motion. Specifically, in addition to the specified start and end points, two more points through which the trajectory passes are set. This divides the overall motion trajectory into three consecutive sections. The motion trajectory of each section is composed of a fivetic Bezier curve and a cubic polynomial. This combination method can ensure that the kinematic parameters of the robot joint are continuous and without sudden changes between each section, thereby significantly improving the operational stability of the robot [16]. The B-5-B mixed polynomial interpolation function is shown in Equation (9).


$$q(t)=\{\begin{array}{c} {}*20c\sum {\mathrm{\backslash nolimits}}_{k=0}^{5}B_{5,k}(u(T))\cdot P_{k}\\ \sum {\mathrm{\backslash nolimits}}_{m=0}^{3}a_{m}(T-T_{1})\\ \sum {\mathrm{\backslash nolimits}}_{k=0}^{5}B_{5,k}(v(T))\cdot Q_{k} \end{array}\begin{array}{c} {}*20ct\in \left[T_{0},T_{1}\right]\\ t\in \left[T_{1},T_{2}\right]\\ t\in \left[T_{2},T_{f}\right] \end{array}$$ (9)

**Fig 3:**
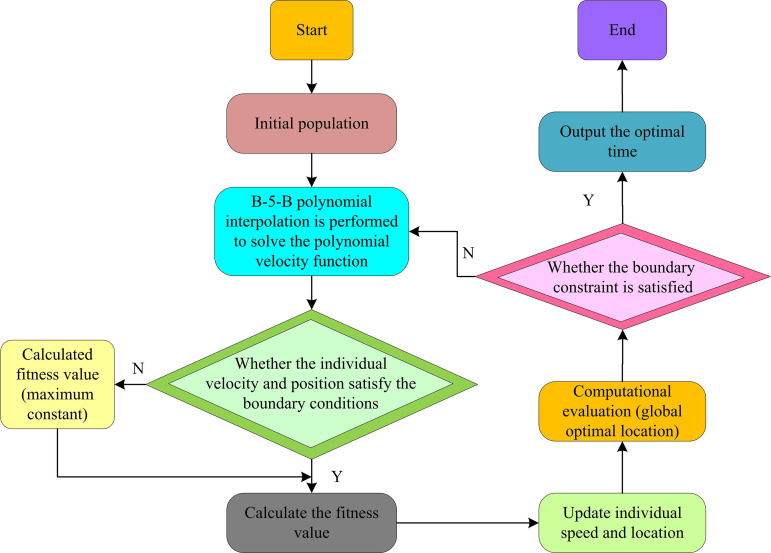
Trajectory optimization flow chart of manipulator based on B-5-B mixed polynomial interpolation function and improved SCSO.

In Equation (9), $T_{1}$ denotes the duration of the action of the control initial Bézier segment. $T_{2}$ denotes the control polynomial segment’s action duration. u(T) and v(T) both denote normalized time parameters. $P_{k}$ and $Q_{k}$ both denote the control point vector of the Bézier segment. $a_{m}$ denotes the coefficients of the cubic polynomial segment. $B_{5,k}(u)$ denotes the fifth degree Bessel basis function. The flowchart of RA trajectory optimization based on B-5-B mixed polynomial interpolation function and improved SCSO is shown in Fig 3.

In Fig 3, before the optimization starts, the position and velocity of the SC group need to be initialized first. The initial values of position and velocity are randomly generated within the joint motion range and velocity range of the RA [17]. For each individual’s position, a B-5-B mixed polynomial interpolation function is used to compute its corresponding polynomial velocity function. Then, each individual’s velocity and position are checked to determine if they are within the range of motion of the RA. If outside the boundary, the great constant of the FV is calculated. If the boundary is not exceeded, the adaptation value of each individual is calculated, including path length, movement time, joint moment, etc. The individual’s position and velocity are updated based on the FV. The existing population’s global ideal position is assessed. The position of the person with the lowest FV is the global ideal position. The updated individual positions and velocities are checked to see if the boundary conditions are satisfied. If they do not satisfy, it is necessary to re-enter the second step for adjustment. If they do, the optimal time is output and the optimization is ended.

### 2.2 RA motion control based on compound control and improved SCSO

**Fig 4:**
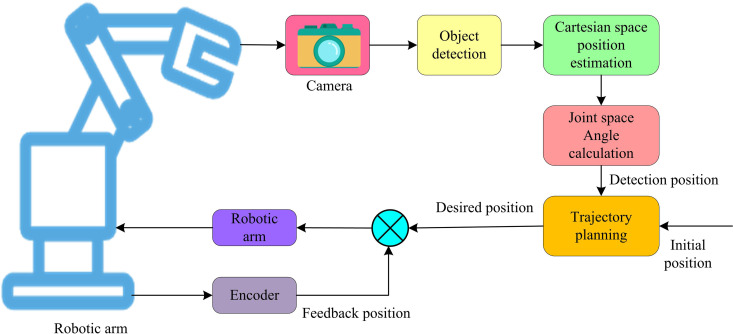
Structure flow of location-based visual servo control system.

Using the enhanced SCSO algorithm to design the RA motion path, this research uses visual guidance to regulate the RA motion, particularly in complex or dynamically changing settings, to further increase the accuracy and adaptability of the RA operation. The structural flow of the position-based visual servo control system is shown in Fig 4.

**Fig 5:**
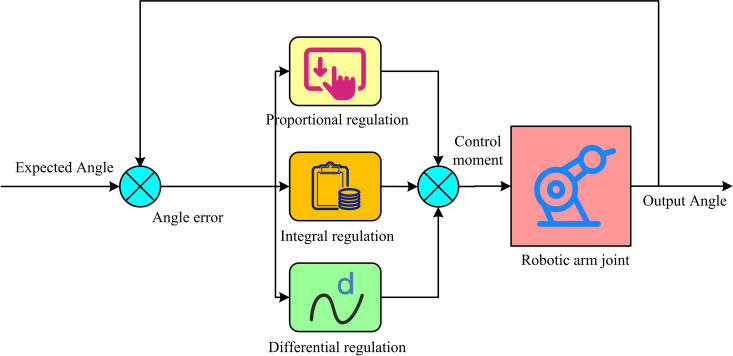
PID control system structure diagram.

In Fig 4, the camera is used to capture the image information of the end-effector of the RA and the target object (TO), and the image acquired by the camera is processed to identify and locate the TO [18]. The position of the TO is converted from the image coordinate system to the Cartesian space coordinate system. The angle to be reached by each joint of the RA is calculated according to the target spatial coordinate system. The angle information is combined with the motion constraints and performance requirements of the RA for trajectory planning. Encoder is mounted on the joints of the RA and used to measure the actual angle or position of the joints. The actual position information of the actuator at the end of the RA is used to compare with the desired position to generate control signals. Using proportional, integral, and differential calculations based on the system error, the PIDC regulates the control quantity. Because of its straightforward design, strong stability, dependable operation, and ease of adjustment, it has emerged as one of the primary technologies for industrial control [19,20]. Therefore, the study introduces the PIDC to precisely control the motion of the RA on the basis of visual guidance. The schematic structure of PID control system is shown in Fig 5.

In Fig 5, the PID control system is a closed-loop control system. The control signal is adjusted in real time by comparing the actual output with the reference input through the feedback loop. Among them, the proportional control controls according to the current deviation value in a certain proportional relationship. The larger the deviation, the stronger the control effect. Integral control adjusts the control action according to the cumulative effect of the deviation, which can eliminate the static error and gradually stabilize the system. Differential control predicts and adjusts the control action according to the trend of the deviation, which can reduce the overshoot of the system and improve the response speed [21]. The expression of the PID control output u(T) of the continuous system, as shown in Equation (10).


$$u(T)=K_{p}e(T)+K_{i}\int_{0}^{T}e(\tau )d\tau +K_{d}\frac{de(T)}{dT}$$ (10)

In Equation (10), e(T) is the error signal. $K_{p}$ is the proportional gain. $K_{i}$ is the integral gain. $K_{d}$ is the differential gain. Among them, the joint space control error signal q(T) is shown in Equation (11).


$$e_{q}(T)=q_{d}(T)-q(T)$$ (11)

**Fig 6:**
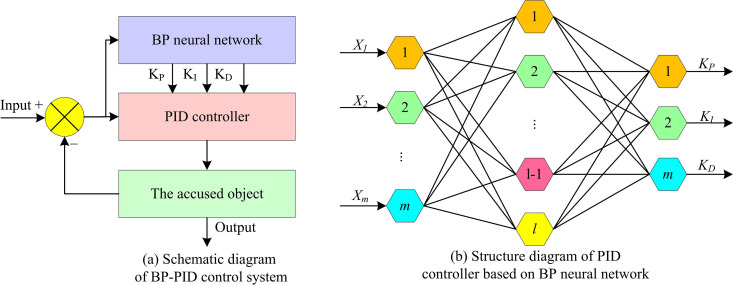
Structure diagram of PIDC based on BPNN.

In Equation (11), $q_{d}(T)$ is the desired joint angle (JA). $q_{d}(t)$ denotes the actual JA fed back by the encoder. The traditional PIDC parameter adjustment depends on experience, which is not only time-consuming and laborious, but also difficult to adapt to the real-time changes of complex dynamic systems. Therefore, the study introduces the BPNN to automatically adjust the PID parameters to achieve a better control effect. The BP-PID controller’s primary function is to automatically adjust the PID parameters based on real-time error signals from the system to precisely control the RA’s movement. In terms of the operation mechanism, the BP-PID controller adopts the online real-time optimization mode. The controller continuously collects the motion state data of the RA at a cycle of 1ms. During each control cycle, the BP neural network quickly calculates and outputs optimized PID parameters based on the most recent deviation information. This enables the controller to promptly respond to changes in the RA’s dynamic characteristics or external disturbances. Fig 6 displays the PIDC’s structural and schematic diagram based on BPNN.

The schematic diagram of the PIDC based on BPNN is displayed in Fig 6(a). By learning the link between the input signal and the intended output, the BPNN optimizes the PIDC and can modify its parameters. The PIDC’s schematic structure based on BPNN is displayed in Fig 6(b). m is the number of nodes in the input layer of the BPNN. l is the number of hidden layer nodes of the BPNN [22]. In this case, the outputs of $K_{P}$, $K_{I}$, and $K_{D}$ in the BPNN-based PID control are displayed in Equation (12).


$$\{\begin{array}{c} {}*20cK_{P}=O_{1}^{s}(T)\\ K_{I}=O_{2}^{s}(T)\\ K_{D}=O_{3}^{s}(T) \end{array}$$ (12)

In BPNNs, the Sigmoid function is usually used as the activation function of the hidden and output layers (OLs) [23]. For the PIDC parameter optimization problem, the Sigmoid function of the OL ensures that the PID parameters output by the neural network are non-negative. The expression of the Sigmoid function of the OL is shown in Equation (13).


$$g(x)=\frac{e^{x}}{e^{x}+e^{-x}}$$ (13)

In neural network based PID control, the choice of error function directly affects the direction of parameter optimization [24]. The performance index function J(φ) is shown in Equation (14).


$$J(\varphi )=\frac{1}{2N}\sum_{i=1}^{N}{\left\|r(T_{i})-y(T_{i})\right\|}^{2}$$ (14)

In Equation (14), φ denotes the parameter vector to be optimized. $r(T_{i})$ denotes the desired output at moment $T_{i}$. $y(T_{i})$ denotes the actual output of moment $T_{i}$ [25]. The computation of the gradient descent update rule is displayed in Equation (15).


$$\varphi_{T+1}=\varphi_{T}-\gamma \nabla_{\varphi }J(\varphi_{T})$$ (15)

**Fig 7:**
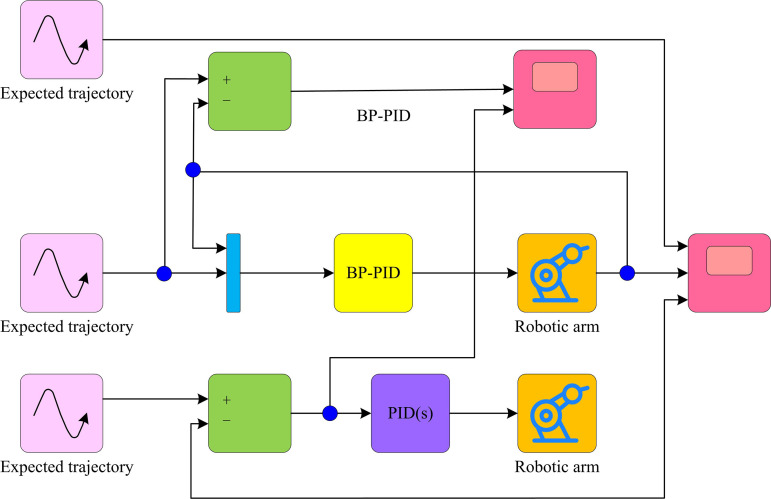
Structure diagram of compound control system based on BP-PID.

In Equation (15), γ denotes the amount of learning. $\nabla_{\varphi }J(\varphi_{T})$ is the gradient of the performance metric function J at $\varphi_{T}$. Fig 7 depicts the schematic layout of the compound control system based on BP-PID.

In Fig 7, the parameters of the BPNN structure are first set and the BP-PID control system is built. At each sampling moment $T_{i}$ of the system operation, the $r(T_{i})$ and the actual output value $y(T_{i})$ of the system are firstly collected, and then the error at that moment is calculated. Subsequently, based on the input error information, the $K_{P}$, $K_{I}$, and $K_{D}$ of the PIDC are calculated. The calculated errors are passed through the BPNN, and the chained partial derivatives of each level are calculated layer by layer [26]. Subsequently, the number of iterations is self-incremented by 1, and the system returns to the sampling step and repeats the above process. Until the end of sampling, the whole system continuously optimizes the control performance during operation. This eventually results in fine control of the regulated RA and guarantees that the output closely follows the intended trajectory.

## 3. Results

To comprehensively verify the scientific validity, effectiveness, and engineering feasibility of the RA motion control model based on compound control and improved SCSO, the study develops a systematic verification system with two dimensions. The test focuses on the performance of the algorithm core. This includes dimensions such as the local development of unimodal functions, the global exploration of hybrid functions, and the processing of nonlinear features of composite functions. The goal is to reveal the technical advantages of the improved algorithm in terms of search accuracy, CS, and stability. On the other hand, the tracking accuracy of the motion trajectories of each joint of the RA, the continuity of end position control, and the dynamic response characteristics are measured under the sinusoidal trajectory input conditions by building a comparative experimental platform for the STM32 microcontroller based on the engineering application scenarios. Combined with the quantitative analysis of kinematic parameters, the technical feasibility of the entire chain from algorithm optimization to control implementation of this model is verified.

### 3.1 Performance of RA motion control algorithm based on compound control with improved SCSO

To verify the effectiveness of the proposed algorithm, four mainstream metaheuristic algorithms are selected as benchmarks for comparison in the study, including whale optimization algorithm (WOA), sparrow search algorithm (SSA), grey wolf optimizer (GWO), northern goshawk optimization (NGO), deep Q-network (DQN), graph neural network (GNN). The single-peak function chosen as f1 to assess the algorithm’s capacity for local exploration is part of the CEC2022 standard test function set, which serves as the basis for the tests. The hybrid function selects f2 and f3 to test the global exploration ability of the algorithm in complex multimode scenarios. The composite function chooses f4 to verify the adaptability of the algorithm to nonlinear features and variable coupling. The search space of all functions is uniformly limited to [−100,100], the population size is 30, and the dimension is 20. The maximum number of iterations is 1000, and the number of independent runs is 30 to eliminate the effect of randomness. The algorithms are comprehensively ranked according to their performance on the three indicators of optimal value (OV), mean, and standard deviation (SD), as shown in Table 1.

**Table 1:** Comparison of evaluation indicators.

Function	Index	Research algorithm	WOA	SSA	GWO	NGO	DQN	GNN
f1	OV	300.29	8611.99	2015.96	3590.08	1671.10	4200.75	3800.32
	MV	557.87	25204.53	7996.13	13235.02	9498.26	8500.41	7200.18
	SD	362.93	9279.16	3665.92	5125.16	4485.03	2100.54	1800.27
f2	OV	2123.21	2096.28	2078.05	2110.00	2126.13	2250.89	2180.47
	MV	2147.93	2156.12	2120.04	2155.02	2170.79	2300.15	2250.62
	SD	13.96	40.68	20.96	30.05	43.01	35.25	28.17
f3	OV	2220.97	2231.70	2228.17	2229.37	2234.26	2350.42	2280.39
	MV	2240.81	2287.71	2243.06	2265.92	2254.15	2400.28	2320.51
	SD	39.02	56.92	29.38	44.95	29.13	42.18	36.25
f4	OV	2956.38	2975.52	2974.79	2975.80	2975.68	3050.21	3020.15
	MV	2996.16	3029.12	3030.75	3038.06	3109.80	3150.33	3080.27
	SD	29.58	38.53	41.25	39.78	105.21	45.12	40.35

**Fig 8:**
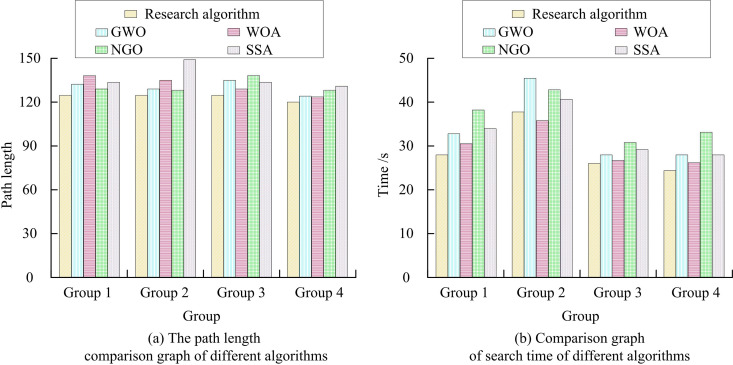
Comparison of path length and search time.

In Table 1, The research algorithm’s OV in the single peak function is 300.29, which is noticeably superior to that of the comparison algorithm. The SD of the study algorithm is 362.93, which is lower than WOA and SSA but higher than NGO. In the hybrid function, the OV of the study algorithm is 2123.21, which is slightly lower than SSA and GWO but higher than WOA and NGO. The mean value (MV) of the study algorithm is 2147.93 and the SD is 13.96, which outperform the alternative methods.. In composite function, the OV of the research algorithm is 2956.38. WOA is 2975.52, SSA is 2974.79, GWO is 2975.80, and NGO is 2975.68. Overall, the research algorithm performs well in terms of global searching ability, stability, and convergence accuracy, and it has a significant advantage in single-peaked and hybrid functions. It shows that the research algorithms have strong adaptability and robustness in dealing with different types of optimization problems. The study conducts several sets of path planning comparison experiments using the RflySim platform in an attempt to confirm the superiority of the suggested method. The experimental setup remains consistent with the above to exclude parameter differences from interfering with the results. For the four randomly generated terrains, each terrain is run independently 10 times to eliminate the effect of chance. The comparison results of path length and search time for different algorithms are display in Fig 8.

The comparison of path lengths for various techniques is displayed in Fig 8(a). Among the compared algorithms, the research algorithm has the strongest path search capability. In the first set of terrains, the path length of the research algorithm is 124. In the second set of terrains, the path length is 127. In the third set of terrains, the path length is 128. In the fourth set of terrains, the path length is 136. This displays that the research algorithm is able to plan shorter paths efficiently in different terrain conditions. The comparison of search times for various methods is demonstrated in Fig 8(b).The research algorithm has the shortest SEARCH TIME in all four groups of terrain. In the first set of terrains, the search time of the research algorithm is 28 seconds. In the second set of terrain, the search time is 37 seconds. In the third set of terrain, the search time is 26 seconds. In the fourth set of terrain, the search time is 23 seconds. It shows that the research algorithm not only has good path planning effect, but also has high search efficiency and can complete the path planning task in a short time.

### 3.2 Application effect of RA motion control technology based on compound control and improved SCSO

**Fig 9:**
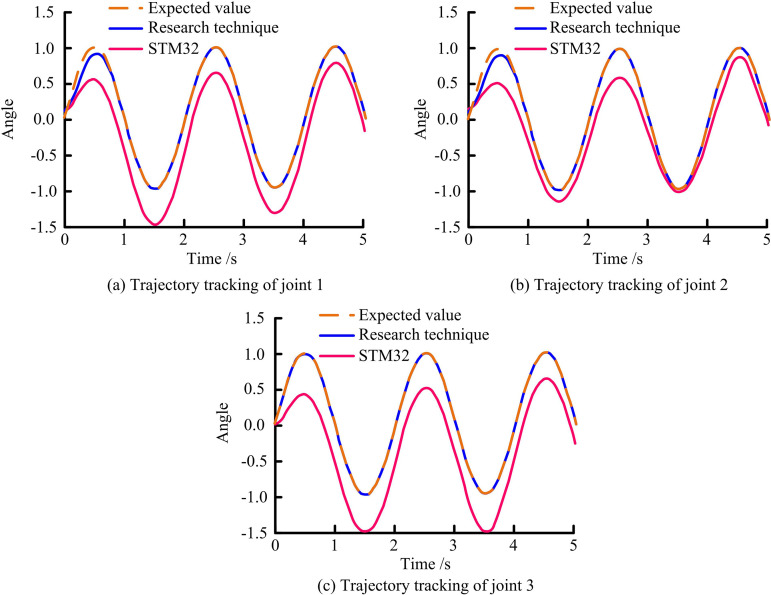
TT of joints 1, 2, and 3.

To verify the application effect of the key technology of RA motion control based on compound control with improved SCSO, the study conducts simulation experiments. The research builds a high-precision simulation experimental environment based on MATLAB/Simulink. Meanwhile, the embedded control system based on the STM32F407ZGT6 microcontroller is adopted as the benchmark comparison platform. This platform integrates a 16-bit ADC sampling module and a high-speed PWM driver unit. These components enable real-time acquisition and precise control of the RA’s motion parameters. In terms of the experimental setup, the study adopts a double closed-loop control architecture. The current loop’s sampling frequency is set to 10 kHz to ensure dynamic response performance. The position loop’s control period is set to 1 ms to ensure control accuracy and stability. To simulate the complex motion requirements of industrial scenarios under the same PID parameters, sinusoidal trajectory input signals with an amplitude of ±30° and a frequency of 1 Hz are applied to each joint of the RA. Sensor measurement errors and external interferences are simulated by introducing Gaussian white noise. To simulate the motion limitations in real application scenarios, the motion ranges of the joints of the RA are strictly set in the experiments. The range of motion of joint 1 is limited to −179° to +179°. The range of motion of joint 2 is limited to −152° to +152°. The range of motion of joint 3, on the other hand, is set at −146° to +146°. The results of TT comparison for joints 1, 2, and 3 are shown in Fig 9.

**Fig 10:**
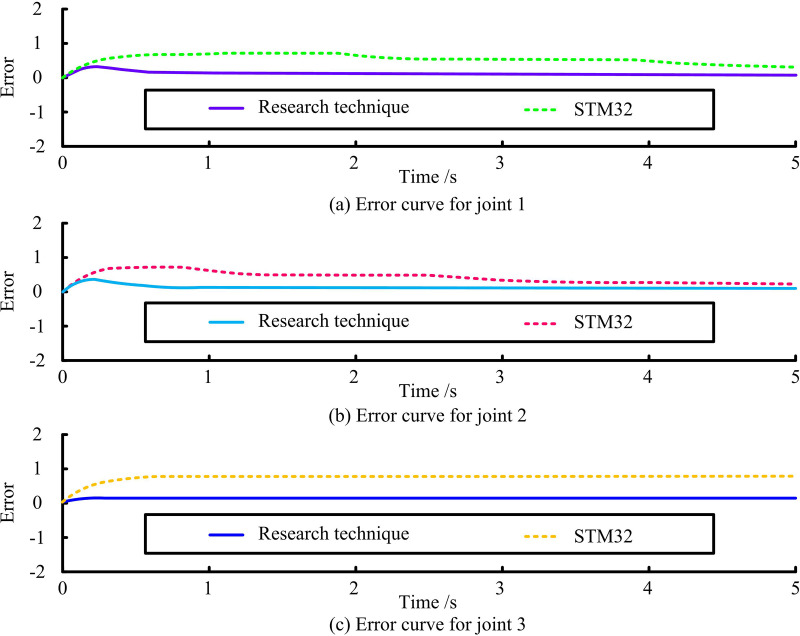
Error contrast curve of two control techniques on joint.

Fig 9(a) shows the TT results of different techniques for joint 1. In the time period from 0 to 1 second, the difference between the proposed technique of the study and the STM32 based control system on the TT of joint 1 is small. The study’s suggested method can precisely track the required value when the time period exceeds one second. On the other hand, the STM32-based control system deviates significantly from the intended value. Fig 9(b) shows the TT results of different techniques for joint 2. The TT results of the proposed technique for joint 2 are slightly different from the desired trajectory. With the passage of time, after 1 second, the proposed technique of the study is able to track the desired value accurately. The STM32 based control system fails to achieve the same tracking accuracy. Fig 9(c) shows the TT results of different techniques for joint 3. The TT results of the proposed technique on joint 1 within 5 seconds of the study are basically in agreement with the desired value. The error comparison curves of the two control techniques for tracking the motion of 3 joints of the RA for these 5 seconds are shown in Fig 10.

**Fig 11:**
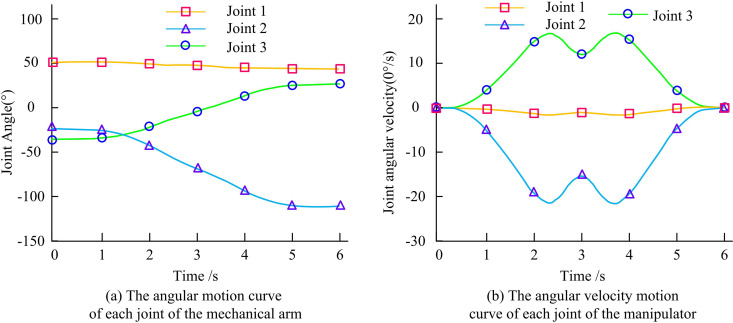
The angular and angular velocity motion curves of the RA joints.

Fig 10(a) shows the error curves of the two control techniques for tracking of joint 1. The error value of the key technology of RA motion control based on compound control with improved SCSO fluctuates around 0.1 during the tracking of RA joint 1 within 0–5 seconds. Moreover, the error value of the STM32-based control system fluctuates around 0.5. Fig 10(b) shows the error curves of the two control techniques for joint 2 tracking. The average error values of the key technology of RA motion control based on compound control with improved SCSO and the control system based on STM32 are about 0.1 and 0.6 during the tracking of the RA joint 2 within 0–5 seconds. Fig 10(c) shows the error curves of the two control techniques for joint 3 tracking. In the time period from 0 to 5 seconds, the error value of the key technology of RA motion control based on compound control with improved SCSO is extremely small and almost negligible, showing extremely high TT accuracy. The error value of the STM32-based control system, on the other hand, fluctuates around 0.4, which is relatively high. Angular velocity is the rate at which angular displacement changes, representing how quickly an object rotates around a fixed axis. In RA motion control, angular velocity directly impacts the smoothness and dynamic response of joint movements. The angular and angular velocity motion curves of the RA joints 1, 2, and 3 are shown in Fig 11.

**Fig 12:**
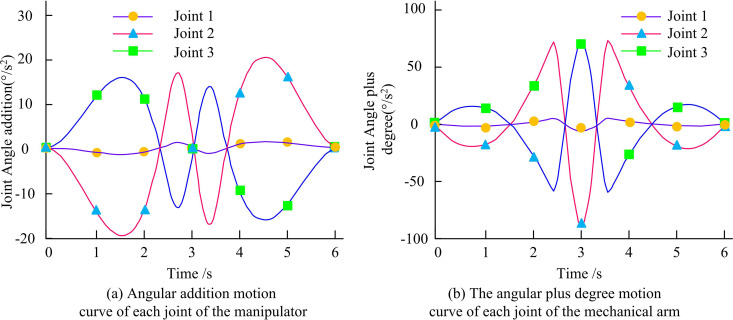
The angular acceleration and angular jerk motion curves of the joint of the manipulator arm.

Fig 11(a) shows the angular motion curves of RA joints 1, 2, and 3. The average JAs of the angular curves of joints 1, 2, and 3 are about 50°, −80°, and 0° based on the key technology of RA motion control with improved SCSO. Fig 11(b) shows the angular velocity motion curves of the RA joints 1, 2, and 3. The average angular velocity curve of the RA motion control key technology based on compound control and improved SCSO for joint 1 is 0°/s. The angular velocity curve of the RA motion control key technology based on compound control and improved SCSO for joint 2 is in M shape. The angular velocity curve of the RA motion control key technology based on compound control and improved SCSO for joint 3 is W-shaped. Angular acceleration refers to the rate of change of angular velocity, reflecting the acceleration or deceleration characteristics of rotational motion. It is directly related to joint torque and influences the dynamic load of the RA. Angular jerk is the rate at which angular acceleration changes. It is a higher-order indicator of motion smoothness. Excessive angular jerk can induce mechanical vibrations that adversely affect the accuracy of end-effector positioning. The angular acceleration and jerk motion curves of RA joints 1, 2, and 3 are shown in Fig 12.

**Fig 13:**
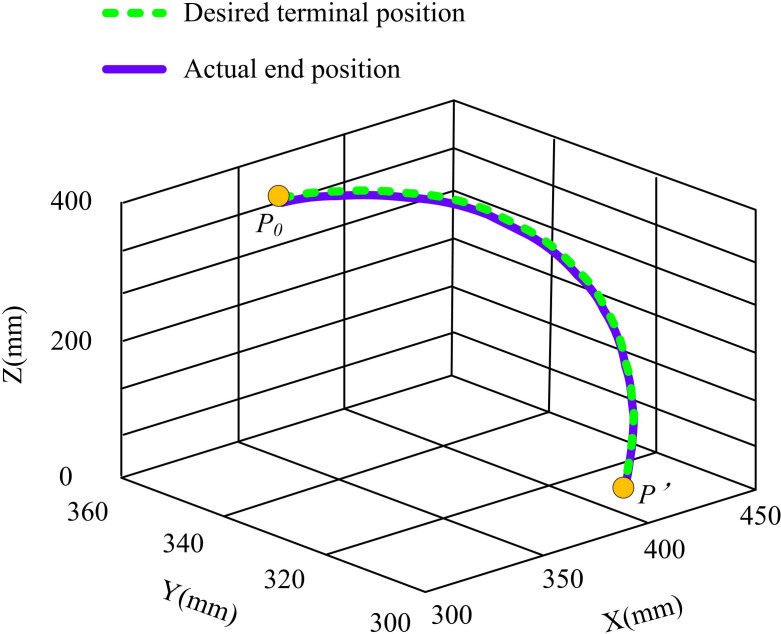
The end position of the RA is actually tracked.

The angular acceleration motion curves for RA joints 1, 2, and 3 are displayed in Fig 12(a). The average angular acceleration of the key technology of RA motion control based on compound control and improved SCSO is 0°/s² for joint 1. For joint 2, the fluctuation range of its angular acceleration is larger. The lowest value is −20°/s², while the highest value is up to 20°/s². Joint 3’s angular acceleration ranges from a low of −17°/s² to a maximum of 17°/s². Fig 12(b) shows the jerk motion curves of RA joints 1, 2, and 3. The angular jerk motion curves of the RA motion control key technology based on compound control and improved SCSO for joints 1, 2, and 3 are characterized by symmetric distribution at the 3rd second. Among them, the average jerk of joint 1 is 0/s^-3^, the minimum jerk of joint 2 is −90/s^-3^, and the maximum jerk of joint 3 is 55/s^-3^. The research technique is used to track the RA’s final position. The results are shown in Fig 13.

In Fig 13, P_0_ denotes the actual end position and P’ denotes the desired end position. When the key technology of RA motion control based on compound control and SCSO is utilized, the actual motion trajectory of the end of the RA shows excellent smoothness throughout the entire motion process. In the whole range, there is no significant jitter or sudden change, which strongly guarantees the smoothness of the movement of the RA. From the perspective of trajectory consistency, the actual trajectory and the desired trajectory of the end position at each time node are highly consistent, showing excellent continuity. This indicates that the RA is able to run in strict accordance with the preset desired trajectory during the movement process, which greatly reduces the errors that may be caused by trajectory deviation. In summary, the proposed control technique performs well in controlling the end position of the RA. It not only realizes smooth and continuous TT, but also significantly improves the control accuracy.

## 4. Discussion and conclusion

### 4.1 Discussion

In the algorithm performance validation, the OV of the proposed algorithm reached 300.29 in the single-peak function f1 test. It significantly outperformed the comparison algorithm with a SD of 362.93, which was lower than that of WOA and SSA, and only higher than that of NGO. This was ascribed to the local search mechanism’s optimization of the enhanced SCSO algorithm, which could more effectively search the GOS in the space of single-peak functions and lessen the likelihood of slipping into the local optimum. N. Yamsani et al. improved the efficiency and reliability of the routing protocol by improving the SCSO algorithm. It proved that the enhanced SCSO algorithm worked well in real-world scenarios. This confirmed the results of the study algorithm’s performance validation. Namely, the improved SCSO algorithm had significant advantages in terms of GSC and stability [27]. In the complex multimodal f2 and f3 function scenarios, the research algorithms had similar convergence ability to the comparison algorithms. This might be due to the special complex structure of the f3 function, which caused the algorithms to not yet reflect significant differences in coping with such complex multimodal scenarios. In the composite function f4 test, the OV of the proposed algorithm was 2956.38, which was better than WOA, SSA, GWO, and NGO. It proved its advantage in dealing with nonlinear features and variable coupling problems. The composite function contained a variety of complex features. The proposed algorithm could be better adapted to this complexity through the organic combination of the compound control strategy and the improved SCSO algorithm. This could effectively adjust the search direction to find a more optimal solution. This was consistent with the findings of Y. Zhang et al.’s investigation on enhanced SCSO and its use in the melting process of aluminum [28].

In the application effect analysis, the average angle of joint 1 was about 50°, and the angular velocity curve was stabilized at 0°/s. It showed that the joint exhibited extremely high stability and precision during the movement, and was suitable for performing tasks requiring high precision. The average angle of joint 2 was approximately −80° and the angular velocity profile was M-shaped. This reflected that the joint could realize dynamic attitude adjustment by switching between rapid acceleration and deceleration during complex trajectory tracking. The flexible motion characteristics allowed Joint 2 to effectively reduce response time and trajectory deviation when dealing with tasks requiring frequent changes in motion direction. Examples included surface grinding and spatial obstacle avoidance. This demonstrated the strong adaptability of the compound control strategy to nonlinear motion scenarios. Joint 3 showed a W-shaped angular velocity variation characteristic at an average Angle of 0°, demonstrating its excellent dynamic response capability. This feature allowed Joint 3 to swiftly perform positive and negative angular velocity alternations during motion task switching. It also enabled agile adjustments to the end posture of the RA and provided a reliable basis for multi-degree-of-freedom collaborative operations. The aforementioned joint motion characteristics corresponded technically with the dynamic response-stability balance theory proposed by B. Zhao et al. in their research on anti-interference control of flexible link manipulators. This further verified the universality of the control strategy under different RA configurations [29]. This was because the suggested method used the BP-PID control method, which improved the system’s dynamic performance and steady state accuracy by automatically adjusting the PIDC’s parameters. Y. Yan et al. used the PID tracking control method to improve the tracking accuracy and stability of the robot in a complex environment. Its echoed the findings of this study in the motion control of RAs. Namely, the performance of the system could be significantly improved by PID control strategy [30].

### 4.2 Conclusion

In the industrial environment, the frequency of RAs is increasing, but their motion control accuracy still needs to be improved. For this reason, the study proposed a RA motion control technique based on compound control with improved SCSO algorithm to enhance its performance in complex motion control. The experimental results revealed that the OV of the proposed algorithm was significantly better than the comparison algorithm in the single-peak function f1 test. It indicated that the algorithm had less fluctuation of results and good stability when solving the single-peak function. In the complex multimodal f2 and f3 function scenarios, the research algorithm could converge more stably to the more optimal solution in multiple iterations. In the composite function f4 test, the OV of the proposed algorithm was equally better than the comparison algorithms WOA, SSA, GWO, and NGO. It proved its superiority in dealing with nonlinear features and variable coupling problems. The joints of the RA using the research technology performed excellently. The average angle, angular velocity, angular acceleration, and angular addition degree of joint 1 all exhibited high stability and accuracy. This indicates that the improved SCSO algorithm can precisely control the motion of joint 1. This enables joint 1 to remain stable during complex motion tasks, providing a solid foundation for the RA’s overall motion control. Joint 2 shows flexibility and adaptability in complex motion. This indicates that the improved SCSO algorithm can flexibly adjust the motion parameters of joint 2 according to different motion requirements. Therefore, it can adapt to various complex motion scenarios and improve the motion flexibility and adaptability of RA. Joint 3 can quickly adjust its posture to adapt to different movement requirements. This demonstrates the improved SCSO algorithm’s ability to respond quickly, adjusting joint 3’s posture according to movement requirements. This improves the RA’s movement efficiency and adaptability. In summary, the RA motion control technique based on compound control and improved SCSO significantly improves the motion control performance of the RA. It provides strong support for high-precision operations in complex industrial environments. This study is mainly conducted in preset dynamic environments, and the robustness of the RA under sudden disturbances is not fully verified. To enhance the algorithm’s online adjustment capability under unknown perturbations, future research can be combined with reinforcement learning. The core of reinforcement learning is that the agent selects an action based on the current environmental state. Integrate deep reinforcement learning organically with the improved SCSO algorithm to construct a dynamic adaptive control framework. By building a highly realistic robotic arm simulation environment and using deep neural networks to extract and encode features of massive information such as the joint state of the robotic arm, environmental interference parameters, and operation task requirements, a high-dimensional state space is formed. The operations such as real-time adjustment of control parameters, online re-planning of motion trajectories, and optimization of obstacle avoidance strategies are abstracted into the action space. On this basis, a multi-level reward mechanism is designed, which not only considers basic indicators such as control accuracy and energy consumption, but also incorporates evaluation criteria for complex scenarios such as response speed to sudden disturbances and obstacle avoidance success rate, driving the agent to continuously learn in the simulation environment and gradually master the optimal control strategy under complex working conditions.

## Supporting information

S1 File Minimal data set definition. (DOCX)
