# Supplementary material for: Key technologies of robotic arm motion control based on compound control and improved SCSO
Source: PLoS One. 2025 Jul 22;20(7):e0328691. doi: 10.1371/journal.pone.0328691 (PMC12282899; doi:10.1371/journal.pone.0328691)
Supplement: S1 File — (DOCX) [file pone.0328691.s001.docx]

The data in Figure 8.

| Function | Number of iterations | Research algorithm | WOA | SSA | GWO | NGO |
| --- | --- | --- | --- | --- | --- | --- |
| f1 | 0 | 50000 | 200000000 | 6000000 | 1000000000 | 50000000 |
|  | 200 | 5000 | 20000 | 25000 | 30000 | 25000 |
|  | 400 | 1000 | 8000 | 22000 | 27000 | 20000 |
|  | 600 | 500 | 5000 | 20000 | 25000 | 20000 |
|  | 800 | 200 | 4000 | 12000 | 20000 | 18000 |
|  | 1000 | 150 | 3000 | 10000 | 8000 | 17000 |
| f2 | 0 | 2300 | 2580 | 2450 | 2680 | 2570 |
|  | 200 | 2210 | 2280 | 2220 | 2340 | 2280 |
|  | 400 | 2205 | 2265 | 2210 | 2320 | 2280 |
|  | 600 | 2205 | 2265 | 2210 | 2300 | 2260 |
|  | 800 | 2205 | 2265 | 2210 | 2290 | 2260 |
|  | 1000 | 2205 | 2265 | 2210 | 2280 | 2260 |
| f3 | 0 | 6000 | 100000 | 100000 | 20000 | 100000 |
|  | 200 | 5000 | 5000 | 5000 | 5000 | 5000 |
|  | 400 | 5000 | 5000 | 5000 | 5000 | 5000 |
|  | 600 | 5000 | 5000 | 5000 | 5000 | 5000 |
|  | 800 | 5000 | 5000 | 5000 | 5000 | 5000 |
|  | 1000 | 5000 | 5000 | 5000 | 5000 | 5000 |
| f4 | 0 | 4600 | 4700 | 4800 | 4900 | 4750 |
|  | 200 | 3000 | 3200 | 3200 | 3250 | 3100 |
|  | 400 | 3000 | 3200 | 3200 | 3200 | 3100 |
|  | 600 | 3000 | 3200 | 3150 | 3200 | 3100 |
|  | 800 | 3000 | 3200 | 3150 | 3200 | 3100 |
|  | 1000 | 3000 | 3200 | 3150 | 3200 | 3100 |

The data in Figure 9(a)

| Time/s | Research algorithm | GWO | NGO | WOA | SSA |
| --- | --- | --- | --- | --- | --- |
| Group 1 | 126 | 135 | 131 | 142 | 134 |
| Group 2 | 127 | 133 | 128 | 137 | 149 |
| Group 3 | 127 | 135 | 133 | 140 | 136 |
| Group 4 | 123 | 131 | 131 | 133 | 134 |

The data in Figure 9(b)

| Time/s | Research algorithm | GWO | NGO | WOA | SSA |
| --- | --- | --- | --- | --- | --- |
| Group 1 | 28 | 33 | 39 | 31 | 34 |
| Group 2 | 38 | 45 | 43 | 36 | 40 |
| Group 3 | 26 | 28 | 31 | 27 | 29 |
| Group 4 | 25 | 28 | 33 | 27 | 28 |

The data in Figure 10 (a)

| Time/s | Research algorithm | Expected value | STM32 |
| --- | --- | --- | --- |
| 0 | 0.0 | 0.0 | 0.0 |
| 1 | 0.0 | 0.0 | -0.5 |
| 2 | 0.0 | 0.0 | -0.5 |
| 3 | 0.0 | 0.0 | -0.2 |
| 4 | 0.0 | 0.0 | -0.2 |
| 5 | 0.0 | 0.0 | -0.1 |

The data in Figure 10

| Time/s | Research algorithm | Expected value | STM32 |
| --- | --- | --- | --- |
| 0 | 0.0 | 0.0 | 0.0 |
| 1 | 0.0 | 0.0 | -0.4 |
| 2 | 0.0 | 0.0 | -0.4 |
| 3 | 0.0 | 0.0 | -0.1 |
| 4 | 0.0 | 0.0 | -0.1 |
| 5 | 0.0 | 0.0 | -0.1 |

Figure 10(c) Trajectory tracking of joint 3

| Time/s | Research algorithm | Expected value | STM32 |
| --- | --- | --- | --- |
| 0 | 0.0 | 0.0 | 0.0 |
| 1 | 0.0 | 0.0 | -0.6 |
| 2 | 0.0 | 0.0 | -0.6 |
| 3 | 0.0 | 0.0 | -0.4 |
| 4 | 0.0 | 0.0 | -0.4 |
| 5 | 0.0 | 0.0 | -0.3 |

The data in Figure 11. (a)

| Time/s | Joint 1 | Joint 2 |
| --- | --- | --- |
| 0 | 0.00 | 0.00 |
| 1 | 0.10 | 0.70 |
| 2 | 0.05 | 0.50 |
| 3 | 0.03 | 0.40 |
| 4 | 0.02 | 0.30 |
| 5 | 0.01 | 0.20 |

The data in Figure 11. (b)

| Time/s | Joint 1 | Joint 2 |
| --- | --- | --- |
| 0 | 0.00 | 0.00 |
| 1 | 0.10 | 0.65 |
| 2 | 0.03 | 0.45 |
| 3 | 0.01 | 0.30 |
| 4 | 0.00 | 0.10 |
| 5 | 0.00 | 0.02 |

The data in Figure 11. (c)

| Time/s | Joint 1 | Joint 2 |
| --- | --- | --- |
| 0 | 0.00 | 0.00 |
| 1 | 0.04 | 0.70 |
| 2 | 0.04 | 0.70 |
| 3 | 0.04 | 0.70 |
| 4 | 0.04 | 0.70 |
| 5 | 0.04 | 0.70 |

The data in Figure 12. (a)

| Time/s | Joint 1 | Joint 2 | Joint 3 |
| --- | --- | --- | --- |
| 0 | 50 | -30 | -35 |
| 1 | 52 | -30 | -40 |
| 2 | 50 | -40 | -20 |
| 3 | 46 | -60 | 0 |
| 4 | 43 | -90 | 10 |
| 5 | 43 | -110 | 25 |
| 6 | 43 | -110 | 30 |

The data in Figure 12. (b)

| Time/s | Joint 1 | Joint 2 | Joint 3 |
| --- | --- | --- | --- |
| 0 | 0 | 0 | 0 |
| 1 | 0 | -5 | 5 |
| 2 | -1 | -20 | 15 |
| 3 | -1 | -15 | -12 |
| 4 | -1 | -20 | 15 |
| 5 | 0 | -5 | 5 |
| 6 | 0 | 0 | 0 |

The data in Figure 13. (a)

| Time/s | Joint 1 | Joint 2 | Joint 3 |
| --- | --- | --- | --- |
| 0 | 0 | 0 | 0 |
| 1 | -1 | -14 | 12 |
| 2 | 1 | -14 | 11 |
| 3 | 1 | -4 | 0 |
| 4 | 2 | 13 | -9 |
| 5 | 1 | 16 | -12 |
| 6 | 0 | 0 | 0 |

The data in Figure 13. (b)

| Time/s | Joint 1 | Joint 2 | Joint 3 |
| --- | --- | --- | --- |
| 0 | 0 | 0 | 0 |
| 1 | 0 | -20 | 20 |
| 2 | 5 | 30 | -25 |
| 3 | -5 | -90 | 85 |
| 4 | 0 | 25 | -25 |
| 5 | -5 | 5 | 15 |
| 6 | 0 | 0 | 0 |

The data in Figure 14

| Time/s | Position /mm | P0 | P’ |
| --- | --- | --- | --- |
| Actual end position | X | 372 | 410 |
|  | Y | 360 | 324 |
|  | Z | 335 | 10 |
| Desired terminal position | X | 373 | 410 |
|  | Y | 360 | 324 |
|  | Z | 336 | 10 |
